# New Insights into the Identification of Metabolites and Cytokines Predictive of Outcome for Patients with Severe SARS-CoV-2 Infection Showed Similarity with Cancer

**DOI:** 10.3390/ijms24054922

**Published:** 2023-03-03

**Authors:** Susan Costantini, Gabriele Madonna, Elena Di Gennaro, Francesca Capone, Palmina Bagnara, Mariaelena Capone, Silvia Sale, Carmine Nicastro, Lidia Atripaldi, Giuseppe Fiorentino, Roberto Parrella, Vincenzo Montesarchio, Luigi Atripaldi, Paolo A. Ascierto, Alfredo Budillon

**Affiliations:** 1 Experimental Pharmacology, Istituto Nazionale Tumori—IRCCS—Fondazione G. Pascale, 80131 Naples, Italy; 2 Melanoma, Cancer Immunotherapy and Development Therapeutics Unit, Istituto Nazionale Tumori—IRCCS—Fondazione G. Pascale, 80131 Naples, Italy; 3 UOC Biochimica Clinica, AORN Ospedali dei Colli—Monaldi—Cotugno—CTO, 80131 Napoli, Italy; 4 Dipartimento di Scienze Mediche Traslazionali, University of Campania “Luigi Vanvitelli”, 80138 Naples, Italy; 5 UOC Fisiopatologia e Riabilitazione Respiratoria, AORN Ospedali dei Colli—Monaldi—Cotugno—CTO, 80131 Napoli, Italy; 6 UOC Malattie Infettive ad Indirizzo Respiratorio, AORN Ospedali dei Colli—Monaldi—Cotugno—CTO, 80131 Napoli, Italy; 7 UOC Oncologia, AORN Ospedali dei Colli—Monaldi—Cotugno—CTO, 80131 Napoli, Italy; 8 Scientific Directorate, Istituto Nazionale Tumori—IRCCS—Fondazione G. Pascale, 80131 Napoli, Italy

**Keywords:** cytokines, metabolites, patient outcome, SARS-CoV-2 infection, cancer

## Abstract

SARS-CoV-2 infection is characterized by several clinical manifestations, ranging from the absence of symptoms to severe forms that necessitate intensive care treatment. It is known that the patients with the highest rate of mortality develop increased levels of proinflammatory cytokines, called the “cytokine storm”, which is similar to inflammatory processes that occur in cancer. Additionally, SARS-CoV-2 infection induces modifications in host metabolism leading to metabolic reprogramming, which is closely linked to metabolic changes in cancer. A better understanding of the correlation between perturbed metabolism and inflammatory responses is necessary. We evaluated untargeted plasma metabolomics and cytokine profiling via ^1^H-NMR (proton nuclear magnetic resonance) and multiplex Luminex assay, respectively, in a training set of a limited number of patients with severe SARS-CoV-2 infection classified on the basis of their outcome. Univariate analysis and Kaplan–Meier curves related to hospitalization time showed that lower levels of several metabolites and cytokines/growth factors, correlated with a good outcome in these patients and these data were confirmed in a validation set of patients with similar characteristics. However, after the multivariate analysis, only the growth factor HGF, lactate and phenylalanine retained a significant prediction of survival. Finally, the combined analysis of lactate and phenylalanine levels correctly predicted the outcome of 83.3% of patients in both the training and the validation set. We highlighted that the cytokines and metabolites involved in COVID-19 patients’ poor outcomes are similar to those responsible for cancer development and progression, suggesting the possibility of targeting them by repurposing anticancer drugs as a therapeutic strategy against severe SARS-CoV-2 infection.

## 1. Introduction

The clinical spectrum of SARS-CoV-2 infection ranges from asymptomatic infection to critical illness leading to hospitalization and intensive care unit (ICU) admission. The most common symptoms observed in infected patients are fever or chills, cough, shortness of breath or difficulty breathing, fatigue, muscle or body aches, headache, loss of taste or smell, sore throat, congestion or runny nose, nausea or vomiting and diarrhea [1].

There is an urgent need for effective therapeutic strategies and for the identification of novel biomarkers associated with poor patient outcomes.

In this context, many researchers are applying -omics techniques to detect mutations, discover drug targets and define the biochemical mechanisms involved in SARS-CoV-2 infection and spread. Therefore, all the efforts provided by -omics-scale investigations and applied to COVID-19 research have been defined with the term COVID-omics [2].

COVID-omics approaches have been so far directed to identify and track new variants, to better understand mechanisms of the disease progression as well as to unveil novel drug targets and vaccines [2,3]. Thus, by genomics and transcriptomics, different SARS-CoV-2 mutations, responsible for the occurrence of new variants, have been identified. For example, proteomics and metabolomics approaches have been applied to body fluids, tissues and cells to understand functional alterations due to virus infection, thus suggesting new disease markers to monitor over time [2]. A lipidomic analysis allowed the identification of COVID-19-associated lipid dysregulation, thus suggesting novel drug targets to prevent coronavirus infection [4]. Recently, multiomics data integration obtained from samples from multiple biological matrices (i.e., bronchoalveolar lavage fluid, blood, throat swabs, cell lines, tissues) has been suggested for a better understanding of the COVID-19 pathophysiological processes, the prediction of patient outcomes, and the definition of new molecular targets for therapeutic interventions [5].

In recent years, our group has focused on the application of cytokine profiling and metabolomics approaches to patients with different diseases, including cancers [6,7,8,9,10,11].

Cytokine-release syndrome (CRS) often develops in patients with a severe form of SARS-CoV-2 infection [12]. This process is referred to a “cytokine storm”, which is induced by macrophages and other innate immune cells; is characterized by the release of proinflammatory cytokines such as IL-1β, IL-6 and TNF-α; and leads to multiple organ failure, widespread damage, and fatal clinical outcomes [13,14,15]. Therefore, identifying the key cytokine networks can enable the development of treatment strategies to block this cytokine storm, such as the cytokine-neutralizing antibodies sarilumab and tocilizumab (targeting the IL-6 receptor), as well as adalimumab and infliximab (targeting TNF-α) [16].

In this context, it is noteworthy that COVID-19 disease and cancer share overlapping inflammatory mechanisms and outcomes [17]. Specifically, the host immune system is involved in cancer development by inducing inflammatory responses [18]. Furthermore, cancer immunity contributes to cancer phenotypes and is associated with compromised immune checkpoints, such as programmed cell death protein 1 (PD-1) or its ligand (PD-L1) [19]. Hence, cancer inflammation favors carcinogenesis, and alterations in several inflammatory cytokines contribute to immune evasion, promoting tumor growth and spread [18]. Some authors have discussed that IL-6 production is enhanced during SARS-CoV-2 infection, as well as during other viral infections associated with the initiation of several cancers, such as hepatitis B virus (HBV), hepatitis C virus (HCV), human immunodeficiency virus (HIV) and human papilloma virus (HPV). Thus, this suggested a potentially direct interaction between SARS-CoV-2 and virus-associated cancers, leading to the hypothesis that IL-6 could bridge the gap between COVID-19 and virus-mediated cancers [20].

Moreover, SARS-CoV-2 infection induces modifications in host metabolism, including pathways related to amino acids, energy generation, and lipids, leading to metabolic reprogramming similar to the metabolic changes contributing to cancer development [21]. Several studies were published regarding the evaluation of the serum levels of metabolites in patients with SARS-CoV-2 infection, which provided evidence that dysregulated metabolites correlated with disease severity [22,23,24,25].

Additionally, since the 1960s, an association between metabolism and immunity has been reported [26] and represents a putative determinant of the antitumor immune response in cancer [27]. For example, IL-6 was found to be able to modulate glucose and lipid metabolism, highlighting the key role of cytokines in host metabolism reprogramming [28,29]. Recent studies have shown several dysregulated metabolic pathways linked to hyperinflammation in SARS-CoV-2 patients with severe infection [30,31].

Hence, since studies identifying novel therapeutic strategies to target inflammation and altered metabolism in cancer are ongoing [32,33,34], a better understanding of the link between perturbed metabolism and inflammatory responses in patients with SARS-CoV-2 infection is necessary.

Therefore, in this study, we evaluated metabolomics using an ^1^H-NMR (proton nuclear magnetic resonance) approach, as well as cytokines, chemokines and growth factors via multiplex Luminex assay, in the plasma of patients with severe SARS-CoV-2 infection, in order to identify novel potential biomarkers to predict patient outcome and novel therapeutic targets. We tested a training set of patients, validated the results in a validation set and then compared the results with those obtained from healthy donor samples.

## 2. Results

### 2.1. Metabolomic Profiling of Plasma Samples from SARS-CoV-2 Patients by ^1^H-NMR

Blood samples from a group of thirty-six patients with severe SARS-CoV-2 infection were collected and classified into two groups, “Exitus” and “Good Prognosis”, on the basis of their outcome, as reported in the Methods section (Table 1).

**Figure 1:**
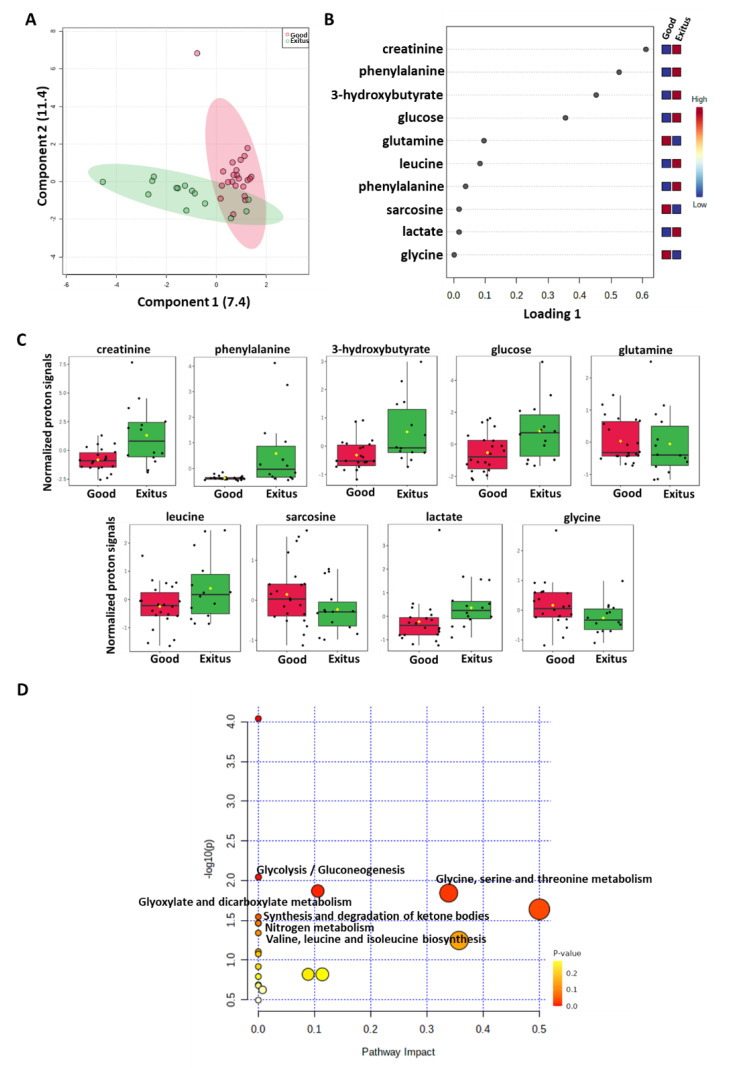
Score plot (**A**) and loading plot (**B**) related to metabolomic profiling of the plasma of SARS-CoV-2 patients. (**C**) Box-whisker plots of the normalized proton signals of the top significant metabolites reported in the loading plot for a given metabolite for patients belonging to “Good Prognosis” and “Exitus” groups. (**D**) The most significant pathways are reported: colors, from yellow to red, indicate increasing levels of statistical significance (in terms of *p* values obtained from the pathway enrichment analysis resulting from the number of metabolites identified and involved in each pathway); size of the nodes indicates pathway impact (a combination of both pathway enrichment results and centrality of each of the matched metabolites within the pathway).

To evaluate whether plasma metabolomic profiling may be informative when predicting their outcome risk, we took advantage of an ^1^H-NMR approach analyzing the collected plasma samples from both patient’s groups. Sparse partial least squares discrimination analysis (sPLS-DA) (18.8% of the total variance) showed that the “Exitus” compared to “Good Prognosis” plasma metabolomics profiles grouped into two different clusters (Figure 1A).

An analysis of the PLS loading was then conducted to identify the metabolites found to be most relevant to the class separation (as reported in the Methods section). As shown in the loading plot reporting the top 10 ^1^H₋NMR signals that were significantly different between the two groups, higher levels of 3-hydroxybutyrate, creatinine, glucose, lactate, leucine and phenylalanine and lower levels of glutamine, glycine and sarcosine were evidenced in the “Exitus” group (Figure 1B,C). Notably, two ^1^H₋NMR signals for phenylalanine were observed, reinforcing the significance of its differential expression between the two patient groups.

Moreover, these metabolites were used to perform a metabolite-set enrichment analysis that highlighted a complex interplay between several different metabolic pathways and metabolites (Figure 1D; Table S1). In detail, aminoacyl-tRNA biosynthesis; glycolysis/gluconeogenesis; glyoxylate and dicarboxylate metabolism; glycine, serine and threonine metabolism; phenylalanine, tyrosine and tryptophan biosynthesis; the synthesis and degradation of ketone bodies; nitrogen metabolism; and valine, leucine and isoleucine biosynthesis emerged to play a role in discriminating the plasma metabolic profiles of the two groups.

A validation set comprising twenty-four patients with severe SARS-CoV-2 infection, classified as “Exitus” or “Good Prognosis”, twelve for each group (Table S2), was also tested, confirming a clear separation in the score plot (Figure S1A). Notably, the validation set the “Exitus” group was also characterized by significant higher levels of 3-hydroxybutyrate, creatinine, glucose, lactate, leucine and phenylalanine and lower levels of glutamine, glycine and sarcosine (Figure S1B).

**Figure 2:**
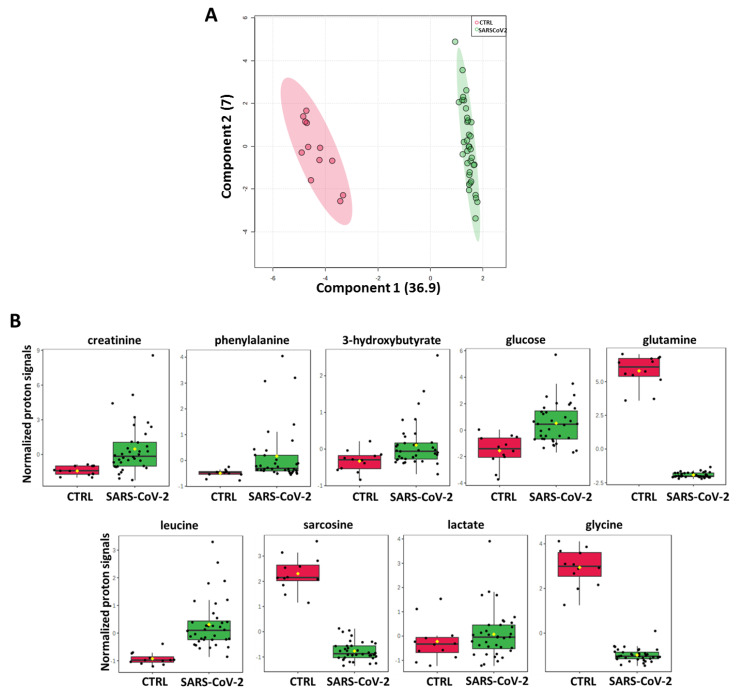
(**A**) Score plot of plasma metabolomic profiling compared SARS-CoV-2-infected patients with an healthy donor cohort (CTRL). (**B**) Normalized ^1^H-NMR proton signals of selected metabolites (same as Figure 1C) in SARS-CoV-2-infected patients compared to CTRL cohort by box–whisker plots.

Furthermore, to add mechanistic insights to our findings, the plasma metabolomics profiling of the first group of thirty-six SARS-CoV-2-infected patients was compared with that of twelve healthy donors (CTRL). As expected, a clear separation between the two groups was evidenced in the score plot (Figure 2A). Interestingly, a targeted analysis of those top ^1^H₋NMR proton signals reported above (Figure 1C), which discriminate “Exitus” vs. “Good Prognosis” patients, similarly distinguish, with same trend, SARS-CoV-2-infected patients from CTRL (Figure 2B).

Next, to establish the optimal cutoff values for the metabolites selected by sPLS-DA, we performed receiver operating characteristic (ROC) curve analysis, which led to area under the curve (AUC) values ranging between 0.63 and 0.83 (Figure S2). Based on the metabolite parameter cutoff values, univariate and multivariate analyses were then conducted to evaluate metabolites potentially associated with patient outcome.

**Figure 3:**
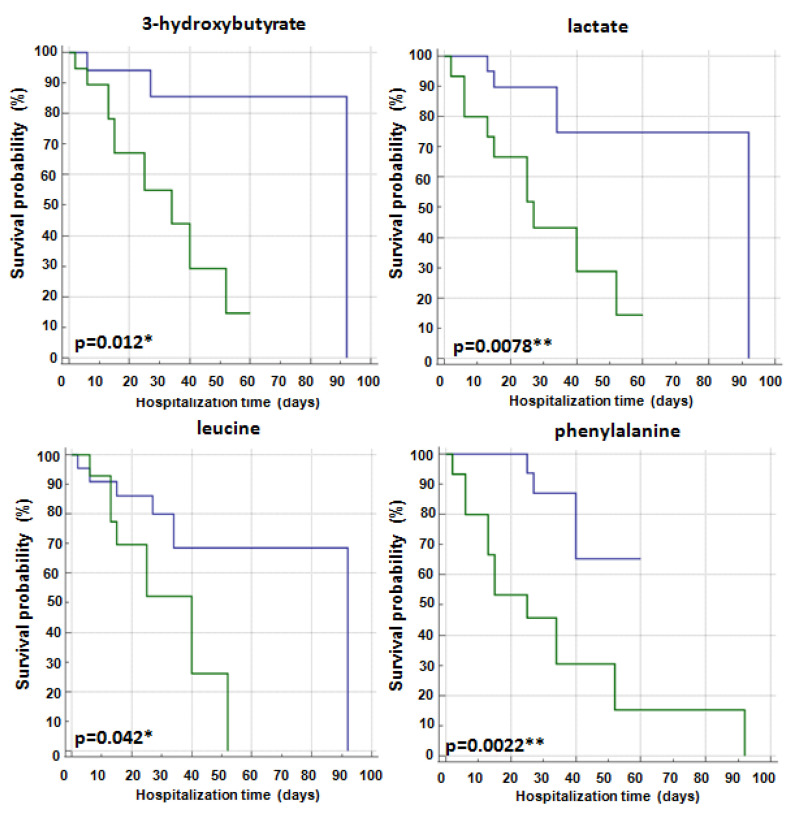
Kaplan–Meier analysis of significant metabolites related to hospitalization time (days) in SARS-CoV-2 patients. Blue line: metabolite levels < cutoff and green line: metabolite levels ≥ cutoff. The significant *p* values are shown in bold. The symbols * and ** represent *p*-value < 0.05 and < 0.01, respectively.

Univariate analysis demonstrated that 3-hydroxybutyrate (*p* = 0.012), lactate (*p* = 0.0078), leucine (*p* = 0.042) and phenylalanine (*p* = 0.0022) predicted SARS-CoV-2-infected patient outcomes (Table 2). As shown by Kaplan–Meier curves related to hospitalization time, lower levels of 3-hydroxybutyrate, lactate, leucine and phenylalanine correlated with a more favorable outcome (Figure 3). Notably, in multivariate analysis, high levels of lactate and phenylalanine retained a significant prediction for survival (Table 2).

### 2.2. Cytokine Profiling of Plasma Samples from SARS-CoV-2 Patients by Multiplex Luminex Assay

**Figure 4:**
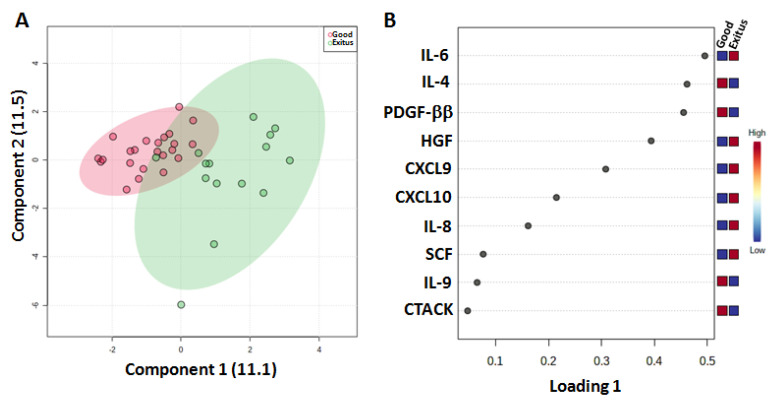
Score plot (**A**) and loading plot (**B**) related to cytokine profiling on plasma of SARS-CoV-2 patients.

Moreover, we evaluated the cytokine levels in thirty-six plasma samples from SARS-CoV-2 patients to verify whether the “Exitus” and “Good Prognosis” groups had different levels of pro- and anti-inflammatory cytokines (Figure S3). The sPLS-DA plot (22.6% of the total variance) showed that the two groups were clearly assembled into two different clusters (Figure 4A). As shown in the loading plot, the “Exitus” group was characterized by higher levels of CXCL9, CXCL10, HGF, IL-6, IL-8 and SCF and lower levels of CTACK, IL-4, IL-9 and PDGF-ββ (Figure 4B and Figure S4).

To determine the optimal cutoff values for the significant cytokines, ROC curve analysis was performed, which revealed AUC values ranging between 0.684 and 0.835 (Figure S5).

**Figure 5:**
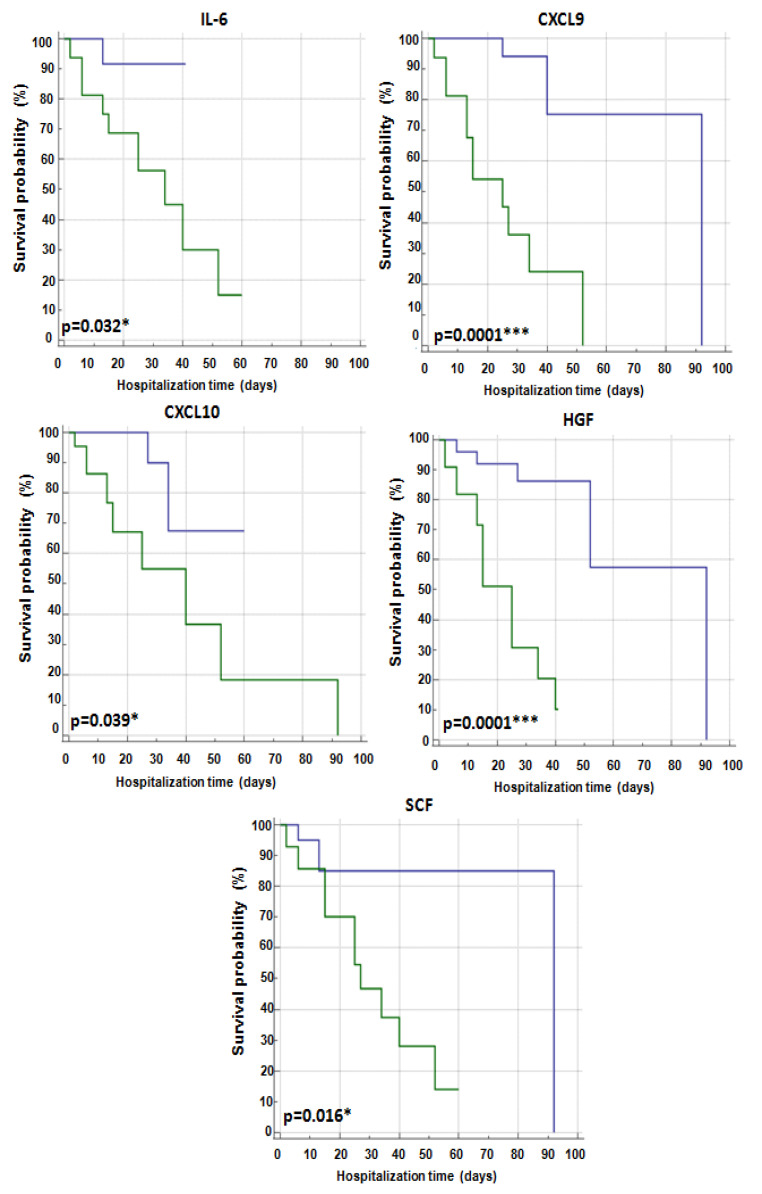
Kaplan–Meier analysis of significant cytokines related to hospitalization time (days) in SARS-CoV-2 patients. Blue line: cytokine levels < cutoff and green line: cytokine levels ≥ cutoff. The significant *p* values are shown in bold. The symbols * and *** represent *p*-value < 0.05 and < 0.0001, respectively.

Univariate analysis demonstrated that CXCL9 (*p* = 0.0001), CXCL10 (*p* = 0.039), HGF (*p* = 0.0001), IL-6 (*p* = 0.032) and SCF (*p* = 0.016) were able to predict patient outcome (Table 3). As shown by Kaplan–Meier curves related to hospitalization time, lower levels of CXCL9, CXCL10, HGF, IL-6, and SCF correlated with a good outcome in these patients (Figure 5). Notably, in multivariate analysis, high HGF levels retained a significant prediction of survival (Table 3).

### 2.3. Identification of Predictive Signatures

**Figure 6:**
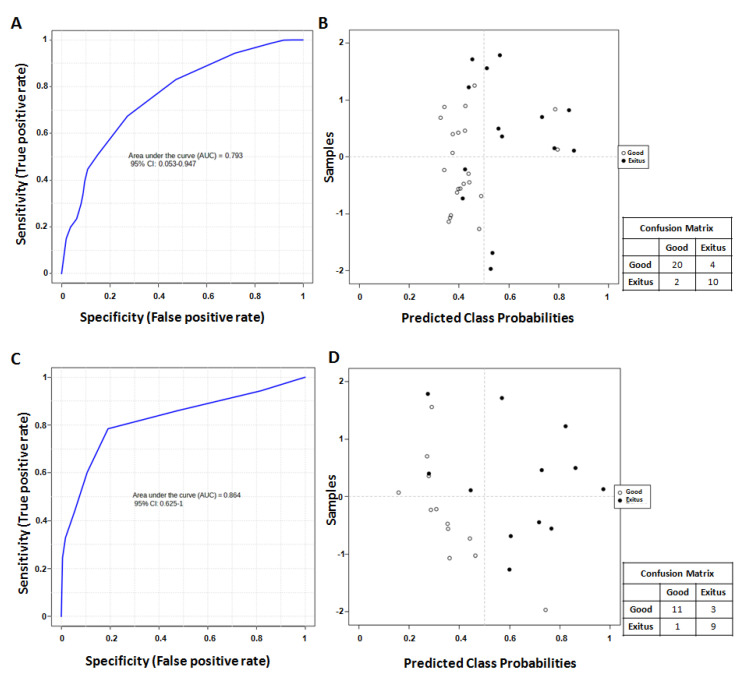
(**A**) Smooth ROC curve related to the combined analysis of HGF, lactate and phenylalanine levels in the first analyzed set. (**B**) Average of predicted class probabilities of each sample across the 100 cross-validations in the first analyzed set. The confusion matrix is reported near the image. (**C**) Smooth ROC curve related to the combined analysis of lactate and phenylalanine levels in the validation set. (**D**) Average of predicted class probabilities of each sample across the 100 cross-validations in the validation set. The confusion matrix is reported near the image.

Finally, we evaluated the predictive capacity of all the possible combinations of the significant metabolites and cytokines selected via univariate analysis (3-hydroxybutyrate, lactate, leucine, phenylalanine, CXCL9, CXCL10, HGF, IL-6, SCF) using a support vector machine (SVM) algorithm. Notably, only the combination of HGF, lactate and phenylalanine levels, the two metabolites and the only cytokine that also had a significant result during multivariate analysis, showed a significant predictive capacity for survival. Specifically, ROC curve analysis performed using the combination of HGF, lactate and phenylalanine levels led to an AUC value equal to 0.793 (95% CI: 0.053–0.947) (Figure 6A). Indeed, the combined analysis of the levels of these analytes classified 20 patients in the “Good Prognosis” group and 10 in the “Exitus” group (Figure 6B) and exhibited a positive predictive value of 83.3% (probability of correct identification of the “Good Prognosis” group) and a negative predictive value of 83.3% (probability of correct identification of “Exitus” group), predicting the outcome of 83.3% (accuracy) of the patients. We also evaluated the predictive capacity of lactate and phenylalanine in the validation set using the same SVM algorithm and verified that the combined analysis of the levels of these two metabolites classified 11 patients in the “Good Prognosis” group and 9 in the “Exitus” group (Figure 6C,D), predicting the outcome of 83.3% (accuracy) of the patients, in the same way as in the first analyzed set.

## 3. Discussion

In the last two years, many studies have been published regarding the evaluation of the serum levels of cytokines and metabolites in patients with severe SARS-CoV-2 infection. Different metabolite/cytokine profiles have been identified, despite the fact that a complete understanding of how host metabolism correlates with inflammatory responses and, above all, with COVID-19 patient’s outcome is still missing.

Our study evaluated untargeted plasma metabolomics and cytokine profiling in a training set of a limited number of patients with severe SARS-CoV-2 infection classified on the basis of their outcome. Notably, the metabolites identified in the training set as able to discriminate patients who died during infection (“Exitus”) from those recovering (“Good Prognosis”), were also confirmed in a validation set comprising additional COVID-19 patients with similar characteristics. Interestingly, these metabolites were also able to discriminate COVID-19 patients from healthy controls, mechanistically suggesting a correlation with poor outcome and confirming somehow the consistency of our data. Similarly, the plasma levels of a large panel of cytokines also clearly distinguish patients based on their outcome. Furthermore, univariate analysis and Kaplan–Meier curves related to hospitalization time showed that lower levels of four metabolites, such as 3-hydroxybutyrate, lactate, leucine and phenylalanine, and of five cytokines/growth factors, such as CXCL9, CXCL10, HGF, IL-6 and SCF, correlated with a good outcome in these patients. However, after the multivariate analysis, only HGF, lactate and phenylalanine retained a significant prediction for survival.

On these bases, taking advantage of SVM, we built a multiple biomarker model, that, by combining the plasma levels of HGF, lactate and phenylalanine, evaluated at the time of hospitalization, was able to correctly classify patients from the training set with their outcome with very good accuracy. Notably, we confirmed this result on the validation set using only lactate and phenylalanine, with similar accuracy, suggesting the critical role of these two metabolites and their potential as combined prognostic biomarkers for hospitalized SARS-CoV-2-infected patients to be evaluated together with conventional clinical parameters.

On the other hand, our data also suggest that the highlighted cytokines and metabolites, particularly those that emerged as statistically significant after univariate analysis, could be related with COVID-19 pathogenesis, thus also representing potential novel therapeutic targets. In this regard, we found similarities between the altered analytes emerging from the present study and the dysregulated cytokines and metabolites that we and many others have found in the peripheral blood of cancer patients being associated with outcome. For example, higher levels of the pro-inflammatory cytokines and chemokines we have selected in the present study, were also found in cancer patients, because of the strict similarity between the inflammatory mechanisms in patients with severe SARS-CoV-2 infection and those with cancer, and the connection with outcome in both patient groups.

In details, in support of our data, other groups reported higher serum levels of IL-6 and IL-8 in patients with SARS-CoV-2 infection at the time of hospitalization. Multivariate analysis showed that IL-6 levels were an independent and significant predictor of disease severity and death in COVID-19 patients [13]. Laing et al. (2020) demonstrated that higher serum levels of CXCL10, IL-6 and IL-10 correlated with disease progression and hospitalization time in SARS-CoV-2-infected patients [14]. Conversely, it is known that IL-6 serum levels correlated with a poor prognosis, tumor burden, survival and progression in different cancers [35], thus being proposed as an anticancer therapeutic target [36]. Recently our group demonstrated that higher levels of IL-6 and CXCL10 were significantly associated with poor disease-free survival in metastatic colorectal cancer (mCRC) patients treated with bevacizumab plus oxaliplatin-based regimens [37]. A recent review article confirmed the significance and mechanism of action of CXCL9, CXCL10 and its receptor (CXCR3) in the development and progression of many tumors [38].

On these bases, and after our experience with cancer patients, we and others proposed to repurpose the monoclonal antibody Tocilizumab, an antagonist of interleukine-6, for COVID-19 therapy, an approach that was actually included in treatment guidelines [16,39,40]. Alongside this successful experience, we suggest that anticancer drugs, which were able to target the analytes we selected in the present study, can be considered as potential treatments against severe SARS-CoV-2 infection.

The activation of AKT signaling, a well-known activated pathway involved in cancers, is involved in the induction of chemokine transcription in response to SARS-CoV-2 infection in a preclinical model, and treatment with the AKT inhibitor GSK690693 markedly reduced CXCL9, CXCL10 and CXCL11 gene expression [41]. Moreover, these data were confirmed in samples from COVID-19-positive individuals displaying marked increases in CXCL9, CXCL10 and CXCL11 transcripts, and an upregulation of components of the AKT signaling pathway was observed via pathway analysis performed on transcriptomic data from these patients [41].

Perreau et al. (2021) showed that in addition to the levels of IL-6, CXCL9 and CXCL10, serum HGF levels were significantly higher in hospitalized ICU patients than in those who were not hospitalized in the ICU. In addition, these authors identified HGF and CXCL13 as biomarkers of disease severity and predictors of ICU admission and death [42]. The increased serum level of HGF, in addition to other analytes, as a marker of COVID-19 severity, was also reported in other studies, although these were conducted using a small number of patients [43,44]. Xu et al. (2020) reported that the concentrations of SCF as well as HGF, IL-6, IL-8, CXCL9 and CXCL10 were significantly higher in fatal than severe and/or mild patients with SARS-CoV-2 infection, which is consistent with our data. Indeed, the levels of SCF, in addition to the levels of HGF, IL-6 and IL-8, were substantially higher in fatal patients during the late stages of the disease, especially at day 14 after diagnosis, and, hence, correlated with death in these patients [45].

Regarding metabolic pathway alterations, in accordance with our data, Meoni et al. evidenced an increase in 3-hydroxybutyrate levels in the plasma of COVID-19 patients compared with healthy controls, and this effect was attributed to an impairment of energetic metabolism [23]. Interestingly, Hwang et al. (2022), reported that the dysregulation of 3-hydroxybutyrate metabolism plays a role in the development of various types of cancer [46]. In this regard, recently, our group highlighted that higher levels of 3-hydroxybutyrate correlated with poor disease-free survival in metastatic colorectal cancer (mCRC) patients treated with bevacizumab plus oxaliplatin-based regimens [37]. The augmented levels of 3-hydroxybutyrate, a component of ketone bodies and an end product of fatty acid β-oxidation, observed in the serum of cancer patients, was suggested to be due to the increase in protein catabolism and fatty acid oxidation needed to fuel cancer cell growth [47]. Similarly, increased energy demand could be related with COVID-19.

Interestingly, targeting metabolic changes through fasting and ketogenic diets seemed capable of inducing beneficial effects in cancer therapy [46], as well as anti-inflammatory and immune-modulating effects in patients with severe SARS-CoV-2 infection, thus preventing and/or modulating cytokine storms [48].

Regarding phenylalanine, its metabolism was indicated as one of the most dysregulated pathways in COVID-19 patients [49]. Correia et al. reported higher levels of phenylalanine in severe COVID-19 patients, suggestive of an altered immune system favoring viral infection [49]. Similarly, the levels of this metabolite are reported as elevated in patients with several cancers [50,51]. In this regard, drugs commonly used in the case of deficiency or mutation of the enzyme phenylalanine hydroxylase (PAH), which catalyzes the hydroxylation of phenylalanine to tyrosine using tetrahydrobiopterin (BH4) and molecular oxygen, can be considered [52].

A systematic review reported that COVID-19 patients with worse outcomes often have higher lactate blood levels compared to those with better outcomes early in the disease’s course [53]. Velavan et al. (2021) monitored lactate concentrations in hospitalized patients and in COVID-19 patients in home quarantine, demonstrating that lactate levels decreased significantly during recovery in hospitalized patients and were significantly higher in hospitalized patients than in home patients, confirming a prognostic role of this metabolite [24]. An additional study also reported high levels of lactate correlating with increased disease severity (from mild to moderate and severe), in a cohort of 52 hospitalized COVID-19 patients [25], in accordance, again, with our data.

High levels of lactate are typically found in cancer patients, also correlating with poor prognosis [54,55]. Taking advantage of an untargeted metabolomics approach, we found higher plasma levels of lactate as well as of phenylalanine compared to healthy controls in large cohorts of melanoma and colorectal cancer patients (Costantini et al. unpublished observation). Lactate excess creates extracellular acidosis, negatively affecting the immune response [56], thus impairing cancer patients’ outcomes as well as COVID-19 recovery. In this regard, based on the experience of lactate dehydrogenase A (LDHA) inhibitors as anticancer therapeutics, these agents were proposed as therapy for COVID-19, because they can affect SARS-CoV-2 replication by reducing glycolysis [56]. Additionally, new lactate-blocking strategies employed in cancer treatment have been evaluated for their potential benefit in COVID-19 in addition to the readily available beta-blockers as an antagonist to lactate [57].

Overall, in the present study, we proposed a prognostic model, using the combined analysis of lactate and phenylalanine levels, to accurately identify, at the time hospitalization, patients with severe SARS-CoV-2 infection with a poor outcome who could benefit from a more intensive follow-up and treatment approach. Moreover, the analytes selected in our study could be used for monitoring disease evolution.

Furthermore, the present study also suggests that the inflammatory mechanisms and the metabolic dysregulation involved in SARS-CoV-2 infection are similar to those responsible for cancer development and progression, also implying the possibility of repurposing anticancer drugs as a therapeutic strategy against SARS-CoV-2 infection.

## 4. Methods

### 4.1. Study Population and Sample Collection

A total of sixty SARS-CoV-2-infected patients (thirty-six in the training set and twenty-four in the validation set) were selected for this study among those hospitalized between 3 March 2020 and 2 May 2020 for respiratory insufficiency at the ‘Azienda Ospedaliera dei Colli Monaldi—Cotugno Hospital’, Italy, but with a negative result for common respiratory pathogens. All patients were hospitalized without a previous positive COVID-19 test. Upon admission to the hospital, a confirmation of SARS-CoV-2 infection was obtained through RT—PCR positivity via an oropharyngeal swab (day onset corresponds to day 0 of hospitalization) following the World Health Organization (WHO) guidelines.

The patients were classified based on their outcome: the “Exitus”, which comprised patients who died during infection, and the “Good Prognosis”, which comprised patients who recovered from COVID-19 (Table 1 and Table S2).

The study was conducted according to the guidelines of the Declaration of Helsinki and was approved on 8 July 2020 by the Ethical Committee of the ‘AORN Ospedali deiColli—Monaldi—Cotugno—CTO, Napoli, Italy’ (approval number AOC-0020053-2020). Informed consent was obtained from all enrolled patients for the use of their biological samples and clinical data for the purposes of clinical research and the study of diseases.

Metabolomics data obtained on plasma samples from twelve healthy donors, nine male and three female, aged >18 years (median = 49; range = 24–60) were obtained within another study from our group (Biocore, IRCCS Pascale Ethical Committee Approval number: 7/14 OSS) and compared with those obtained within the current study.

### 4.2. Plasma ^1^H-NMR Spectroscopy

The plasma samples obtained from the SARS-CoV-2 patients were prepared for NMR analysis by mixing 330 μL of plasma with 300 μL of PBS (containing 10% *v/v* D_2_O) and 70 μL of reference standard D_2_O solution containing 0.1 mM sodium 3-trimethylsilyl [2,2,3,3-2H4] propionate (TSP). They were then inserted into an NMR tube. All the spectra were recorded using a Bruker Avance 600 NMR spectrometer operated at a 599.97 MHz ^1^H resonance frequency and equipped with a cryoprobe. To attenuate the broad NMR signals from slowly tumbling molecules due to lipids and proteins, a standard Carr—Purcell—Meiboom—Gill (CPMG) pulse sequence was used to record the ^1^D spin–echo spectra. To suppress the water peaks, the CPMG presaturation pulse sequence was used using the equation *-RD-90°-(t-180°-t) n—ACQ*, where *RD* is the relaxation delay of 2 s; 90° and 180° represent the pulses that trip the magnetization vector; *t* is the spin–echo delay; n represents the number of loops; and *ACQ* is the data acquisition period. In our experiment, the data points were acquired using 256 transients.

### 4.3. NMR Data Processing

All of the ^1^H-NMR spectra were manually phased and baseline-corrected and referenced to the CH_3_ resonance of TSP at 0 ppm. The spectral 0.50–8.60 ppm region of ^1^H-NMR spectra was integrated in buckets of 0.04 ppm using the AMIX package (Bruker, Biospin, Ettlingen, Germany). In detail, we excluded the water resonance region (4.5–5.2 ppm) during the analysis and normalized the bucketed region to the total spectrum area using Pareto scaling and the MetaboAnalyst v5.0 tool [58].

### 4.4. Pathway Analysis of Significant Metabolites

Pathway analysis of the modulated metabolites was performed using the “Enrichment Functional Analysis” module in the Metaboanalyst v5.0 tool [58]. In detail, we calculated the centrality through pathway impact, a combination of the centrality and pathway enrichment results. Metabolites were selected by evaluating both VIP values of >1 in class discrimination and correlation values of >0.8. Moreover, the Homo sapiens pathway library was chosen and analyzed using Fisher’s exact test for overrepresentation and relative betweenness centrality for pathway topology analysis.

### 4.5. Cytokinome Evaluation

A large panel of cytokines, chemokines and growth factors was evaluated in thirty-six plasma samples from COVID-19 patients using the Bio-Plex assay, which contains dyed microspheres conjugated with a monoclonal antibody highly specific for a target protein. The method was carried out according to the manufacturer’s instructions (Bio-Plex Bio-Rad) to assess the cytokine concentrations. In detail, we used the Bio-Plex Pro™ Human Cytokine Screening Panel, 48-Plex, which consists of assays for the measurement of β-NGF, CCL2 (MCP-1), CCL3 (MIP-1*α*), CCL4 (MIP-1*β*), CCL7 (MCP-3), CCL11 (Eotaxin), CTACK (CCL27), CXCL1 (GRO-α), CXCL9 (MIG), CXCL10 (IP-10), CXCL12 (SDF-1α), FGFbasic, G-CSF, GM-CSF, HGF, IFN-α2, IFN-γ, IL-1α, IL-1*β*, IL-1ra, IL-2, IL-2Rα, IL-3, IL-4, IL-5, IL-6, IL-7, IL-8, IL-9, IL-10, IL-12 (p40), IL-12 (p70), IL-13, IL-15, IL-16, IL-17, IL-18, LIF, M-CSF, MIF, PDGF-*ββ*, RANTES, SCF, SCGF-β, TNF-*α*, TNF-β, TRAIL and VEGF levels.

Protein levels were determined using a Bio-Plex array reader (Luminex, Austin, TX, USA) that quantifies multiplex immunoassays in a 96-well format with very small fluid volumes. The analyte level was calculated using a standard curve with software provided by the manufacturer (Bio-Plex Manager 4.0 Software).

### 4.6. Data Processing and Statistical Analysis

The sparse partial-least-squares discriminant analysis (sPLS-DA) algorithm was applied to explain the maximum separation between the defined class samples in the data (metabolites and cytokines). Score and loading plots were used to highlight and assess the role of X-variables (NMR signals) in the classification models and, hence, to prioritize the discriminating peaks for identification. The NMR signals were compared with reference spectra from the HMDB database [58].

Moreover, the nonparametric Mann—Whitney U test was used to evaluate differences between cytokine concentrations (expressed as pg/mL) in the “Exitus” and the “Good Prognosis” groups. One asterisk (*) indicates differences with *p* < 0.05, two asterisks (**) indicate differences with *p* < 0.01, and three asterisks (***) indicate differences with *p* < 0.0001. The statistical program Prism 6 (GraphPad Software, San Diego, CA, USA) was employed.

Receiver operating characteristic (ROC) curves were calculated for metabolites and cytokines that were found to be significantly correlated with patient outcome using the Biomarker Analysis tool on the Metaboanalyst v5.0 tool [58]. The area under the curve (AUC) was used to assess accuracy. The 95% confidence intervals (CIs) were calculated to compute optimal cutoffs for any given feature (significant metabolites and cytokines).

The Cox regression model was used to assess the role of the cutoff for metabolite and cytokine parameters in predicting patient outcome via MedCalc statistical software (https://www.medcalc.org accessed on 23 July 2022). Hazard ratios (HR) were derived from Cox regression analysis, and their 95% confidence intervals (95% CI) were calculated using the proportional hazard model. Univariate analysis assessed the correlation of metabolites and cytokines with patient outcome.

Multivariate analysis was performed using MedCalc software (https://www.medcalc.org) according to a backward elimination of factors showing a *p* value less than 0.05 in the univariate analysis. In all statistical tests, a *p* value less than 0.05 was considered significant.

Using the module “Biomarker Analysis” in the Metaboanalyst 5.0 tool [58], we performed various biomarker analyses based on ROC curves for multiple biomarkers using a support vector machine (SVM) algorithm. In this way, some biomarker models were tested, and some sample predictions were performed. One hundred cross-validations (CVs) were performed to produce a smooth ROC curve, and the results were averaged to generate the plot. Moreover, the average of the predicted class probabilities of each sample across the 100 cross-validations was produced. Since the algorithm uses a balanced subsampling approach, the classification boundary was located at the center (x = 0.5, the dotted line).

## Figures and Tables

**Table 1:** Clinical data related to thirty-six patients diagnosed with SARS-CoV-2 enrolled in this study.

Study Population	ALL Patients (36)	Good Prognosis (22)	Exitus (14)
Female/Male, *n* (%)	11 (31%)/25 (69%)	8 (36.4%)/14 (63.6%)	3 (21.4%)/11 (78.6%)
Median Age (Range)	64 (29–81)	64 (29–75)	69 (57–81)
Median P/F (Range)	190 (60–378)	241 (60–378)	123 (68–300)
Subjects without comorbidity, *n* (%)	11 (31%)	5 (45%)	6 (55%)
Subjects with ONE comorbidity, *n* (%)	13 (36%)	8 (62%)	5 (38%)
Subjects with TWO comorbidities, *n* (%)	7 (19%)	5 (71%)	2 (29%)
Subjects with THREE comorbidities, *n* (%)	5 (14%)	3 (60%)	2 (40%)
Diabetes, *n*	6	5	1
Hypertension, *n*	20	13	7
Chronic Pulmonary Disease, *n*	7	4	3
Chronic Renal Disease, *n*	5	3	2
Cancer, *n*	4	2	2

**Table 2:** Univariate and multivariate analyses of metabolites for hospitalization time and patient outcome.

	Univariate	Multivariate
Metabolite	HR (95% CI) *p* Value	HR (95% CI) *p* Value
3-hydroxybutyrate level (≥−0.292 nps vs.<−0.292 nps)	4.10 (1.36–12.39) *p* = 0.012 *	1.55 (0.66–5.44) *p* = 0.23
lactate level (≥−0.0324 nps vs. <−0.0324 nps)	4.65 (1.50–14.42) *p* = 0.0078 **	4.71 (1.27–17.30) *p* = 0.020 *
leucine level (≥0.032 nps vs. <0.032 nps)	3.32 (1.04–10.54) *p* = 0.042 *	1.76 (0.67–6.94) *p* = 0.35
phenylalanine level (≥−0.345 nps vs. <−0.345 nps)	6.14 (1.92–19.59) *p* = 0.0022 **	5.81 (1.57–21.48) *p* = 0.0084 **

HR: hazard ratio; CI: confidence interval; nps: normalized values of the proton signals. Significant *p* values are reported in bold. The symbols * and ** represent *p*-value < 0.05 and <0.01, respectively.

**Table 3:** Univariate and multivariate analyses of cytokines for hospitalization time and patient outcome.

	Univariate	Multivariate
Cytokines	HR (95% CI) *p* Value	HR (95% CI) *p* Value
CXCL9 level (≥1180 pg/mL vs.<1180 pg/mL)	11.38 (3.48–37.73) *p* = 0.0001 ***	7.36 (1.37–39.54) *p* = 0.42
CXCL10 level (≥ 1030 pg/mL vs.<1030 pg/mL)	3.25 (1.06–9.96) *p* = 0.039 *	2.26 (0.97–5.41) *p* = 0.34
HGF level (≥1170 pg/mL vs. <1170 pg/mL)	14.45 (3.87–53.95) *p* = 0.0001 ***	6.71 (1.39–32.36) *p* = 0.0022 **
IL-6 level (≥20 pg/mL vs. <20 pg/mL)	4.07 (1.13–14.69) *p* = 0.032 *	1.84 (1.06–3.01) *p* = 0.22
SCF level (≥ 189 pg/mL *vs.<* 189 pg/mL)	3.99 (1.29–12.34) *p* = 0.016 *	2.56 (1.20–4.54) *p* = 0.45

HR: hazard ratio; CI: confidence interval; nps: normalized values of the proton signals. Significant *p* values are reported in bold. The symbols *, ** and *** represent *p*-value < 0.05, <0.01 and <0.0001, respectively.

## Data Availability

Raw data related to Figure 1 and Figure 4 are available at https://gbox.garr.it/garrbox/index.php/s/FdjieMOCUIYM02a, accessed on 21 July 2022.

## Supplementary Materials

The supporting information can be downloaded at: https://www.mdpi.com/article/10.3390/ijms24054922/s1.
