# Supplementary material for: New Insights into the Identification of Metabolites and Cytokines Predictive of Outcome for Patients with Severe SARS-CoV-2 Infection Showed Similarity with Cancer
Source: Int J Mol Sci. 2023 Mar 3;24(5):4922. doi: 10.3390/ijms24054922 (PMC10003544; doi:10.3390/ijms24054922)
Supplement: Supplementary file 1 [file ijms-24-04922-s001.zip › ijms-2046457-supplementary.pdf]

**Table S1.**Enriched molecular function analysis on significant metabolites in the plasma metabolome of patients

| PathwayName | Metabolites | p |
|---|---|---|
| Aminoacyl-tRNA biosynthesis | glycine, glutamine, leucine, phenylalanine | 9,12E-01 |
| Glycolysis / Gluconeogenesis | glucose, lactate | 0.0090641 |
| Glyoxylate and dicarboxylate metabolism | glycine, glutamine | 0.013584 |
| Glycine, serine and threonine metabolism | glycine, sarcosine | 0.014417 |
| Phenylalanine, tyrosine and tryptophan biosynthesis | phenylalanine | 0.023046 |
| Synthesis and degradation of ketone bodies | 3-hydroxybutyrate | 0.028734 |
| Nitrogen metabolism | glutamine | 0.034392 |
| Valine, leucine and isoleucine biosynthesis | leucine | 0.04562 |

**Table S2.** Clinical data related to twenty-four patients diagnosed with SARS-CoV-2 and enrolled in validation set.

| StudyPopulation | ALL Patients (24) | GoodPrognosis (12) | Exitus (12) |
|---|---|---|---|
| Female/Male, n (%) | 3(12.5%)/21(87.5%) | 1(8.3%)/11(91.7%) | 2(16.7%)/10(83.3%) |
| Median Age (Range) | 69(36-92) | 71(37-76) | 65(36-92) |
| Median P/F (Range) | 185(82-428) | 285 (185-428) | 117(82-276) |
| Subjectswithoutcomorbidity, n (%) | 8(33.3%) | 3(25%) | 5(41.7%) |
| Subjects with ONE comorbidity, n (%) | 5(20.8%) | 2(16.7%) | 3(25%) |
| Subjects with TWO comorbidities, n (%) | 8(33.3%) | 5(41.7%) | 3(25%) |
| Subjects with THREE comorbidities, n (%) | 3(12.5%) | 2(16.7%) | 1(8.3%) |
| Diabetes, n | 4 | 2 | 2 |
| Hypertension, n | 14 | 9 | 5 |
| ChronicPulmonaryDisease, n | 7 | 5 | 2 |
| ChronicRenalDisease, n | 1 | 0 | 1 |
| Cancer, n | 3 | 1 | 2 |

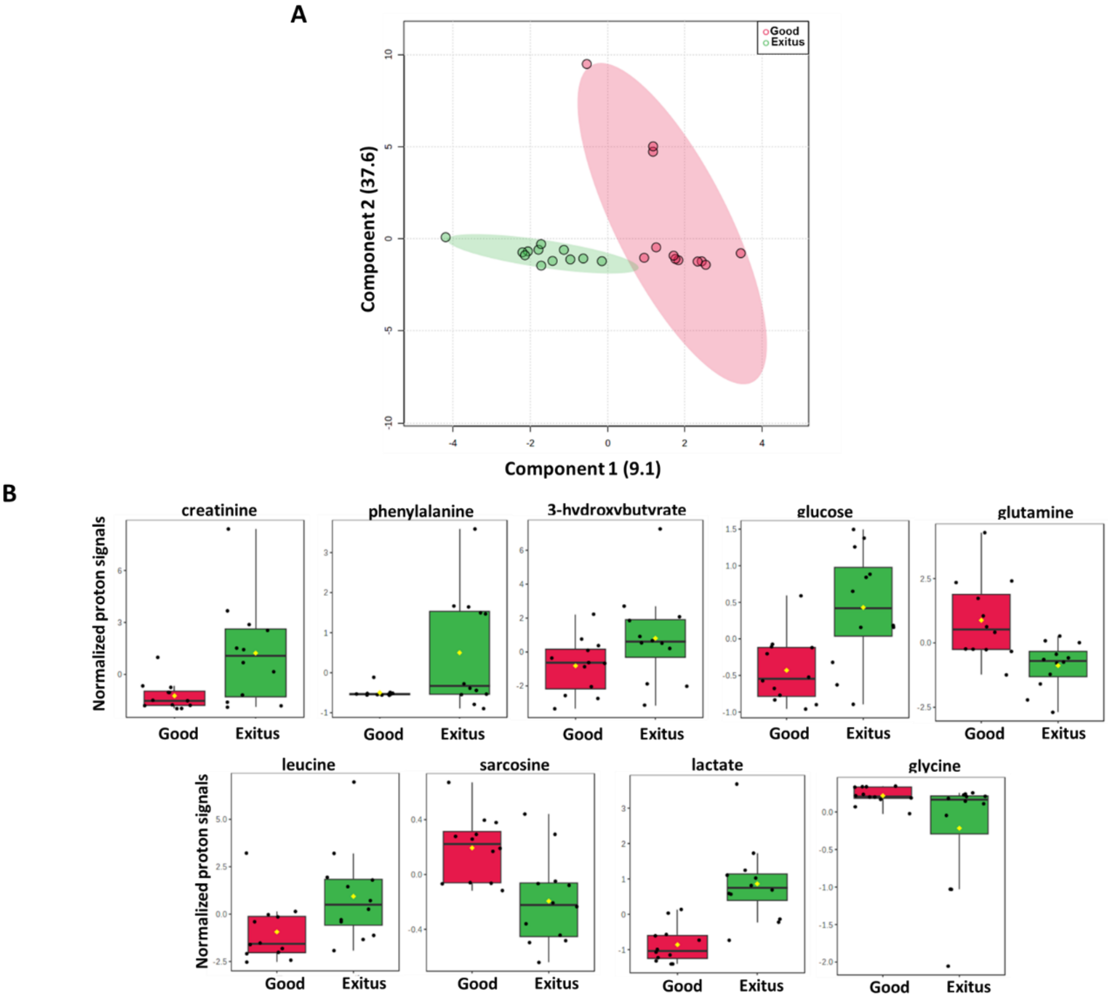


**Figure S1.** (A) Score plot related to metabolomic profiling of the plasma of SARS-CoV-2 patients enrolled in validation set. (B) Box-whisher plots of the normalized proton signals of the selected metabolites reported in Figure1C analyzed in Good Prognosis and Exitus Groups from the validation set.

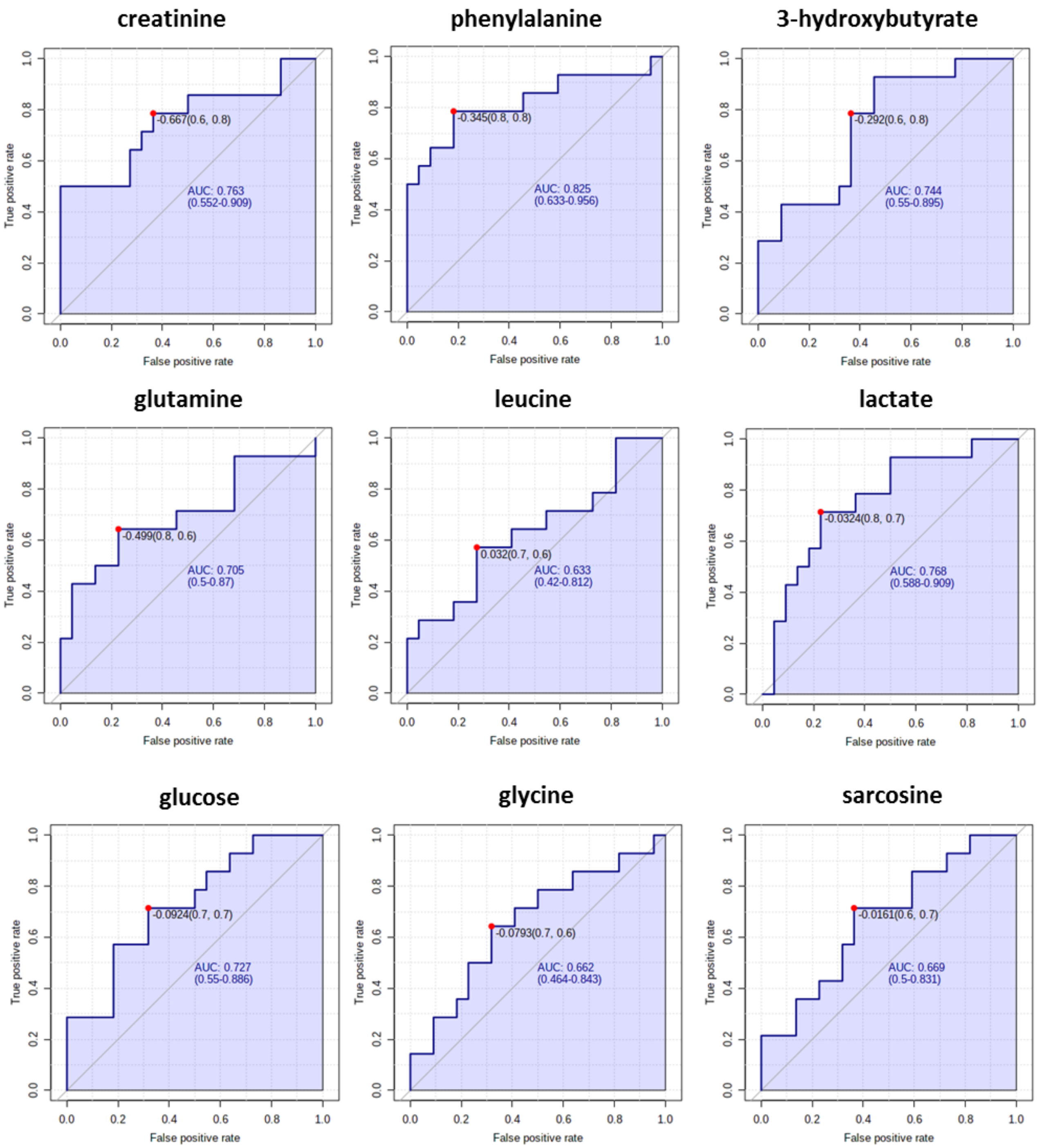


**Figure S2.**ROC curves evaluated on the metabolites that are resulted significant in the sera of patients subdivided between Exitus Group and Good Prognosis Group. The cutoff values are evidenced by red circles

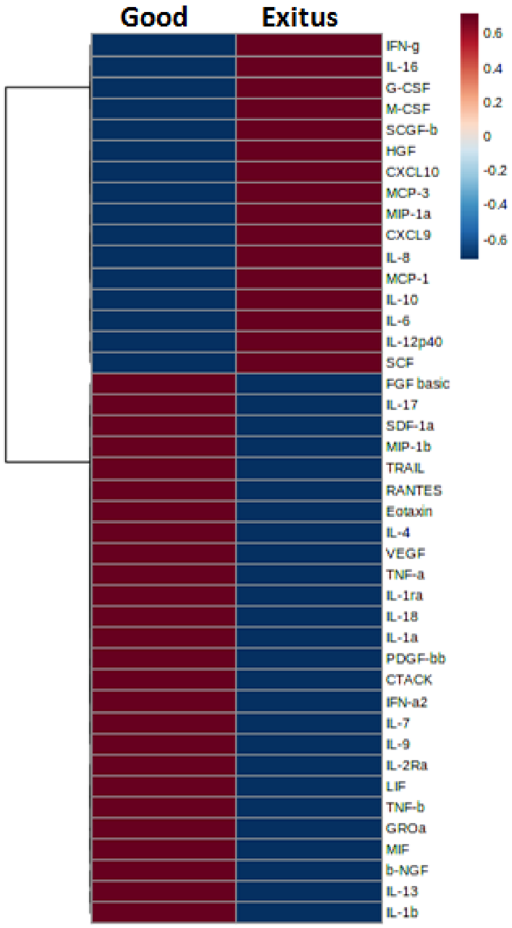


**Figure S3.** Heatmap representation in which it is possible to see what cytokines present higher and lower levels (shown in red and blu, respectively) in Exitus group respect to Good prognosis group.

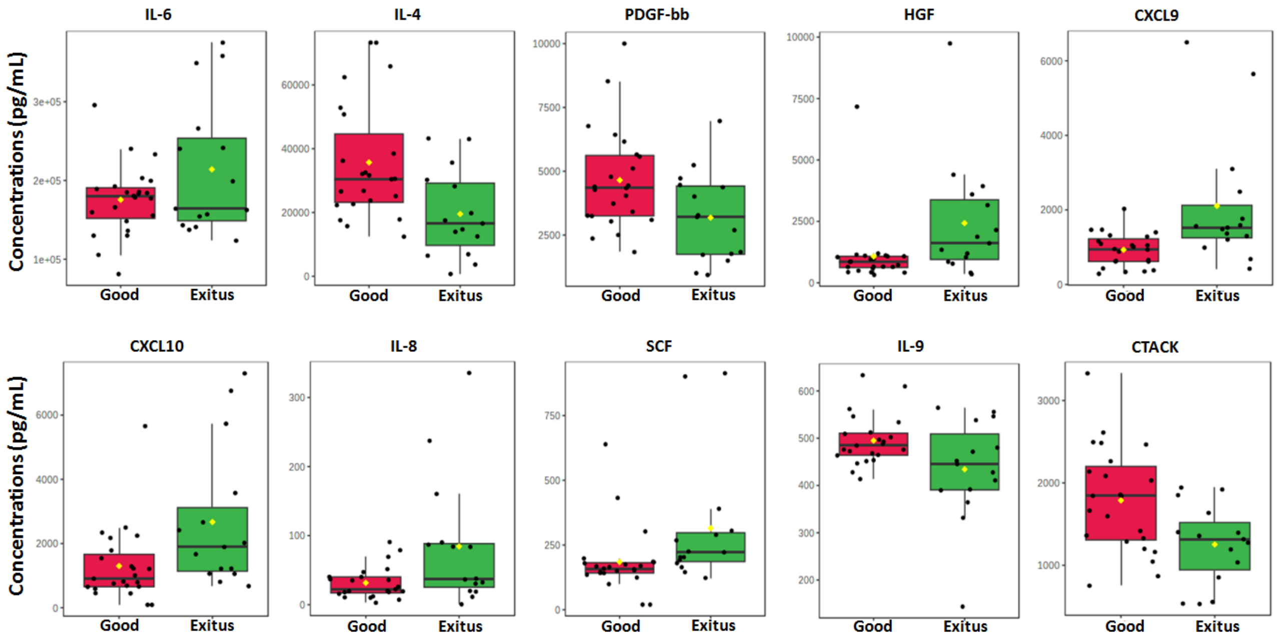


**Figure S4.** We report the top 10 cytokines present in loading plot in Figure 4B by box-whisher plots. Each point indicates the concentration of a given cytokine in each patient belonging to Good Prognosis Group and Exitus Group.

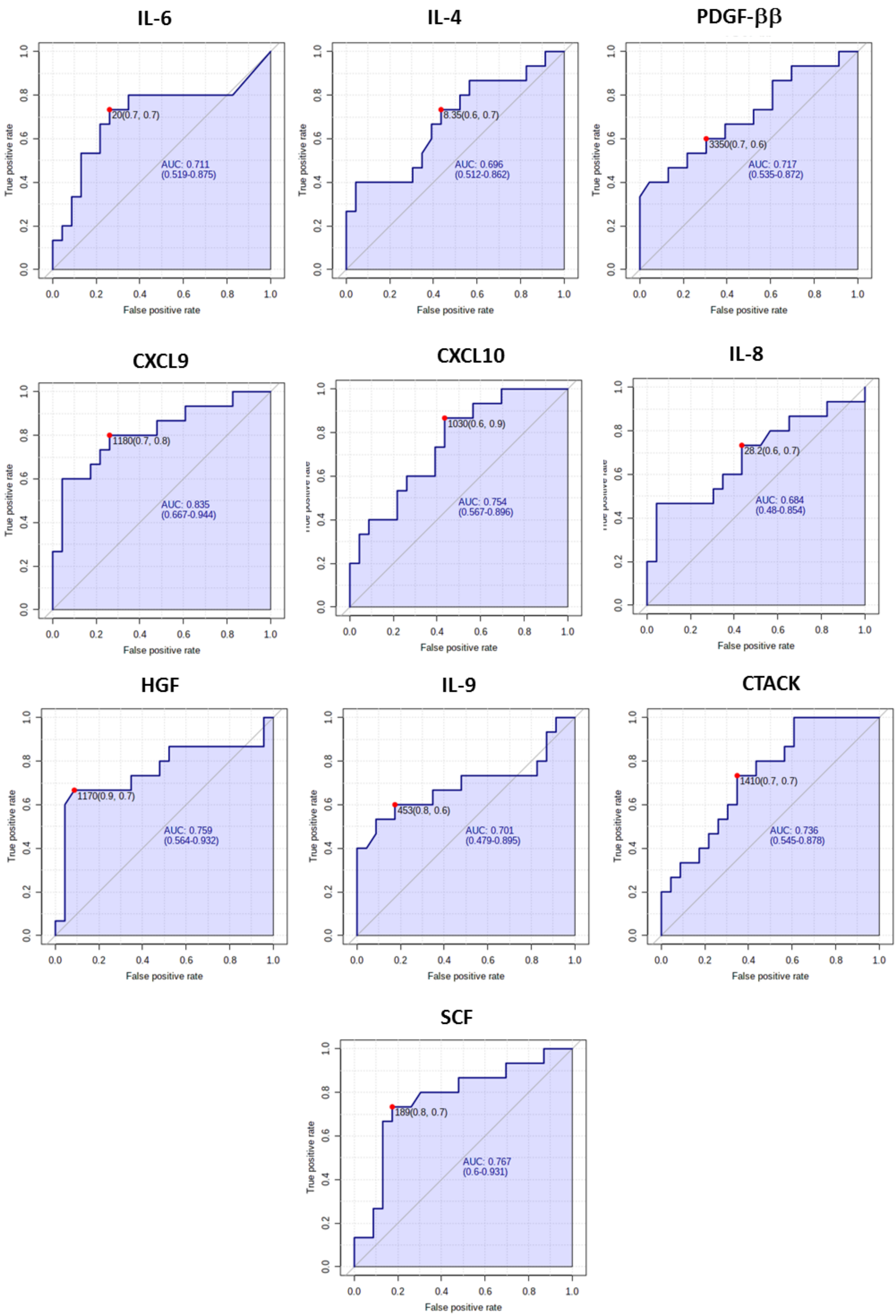

IL-6
20(0.7, 0.7)
AUC: 0.711
(0.519-0.875)
IL-4
8.35(0.6, 0.7)
AUC: 0.696
(0.512-0.862)
PDGF-ββ
3350(0.7, 0.6)
AUC: 0.717
(0.535-0.872)
CXCL9
1180(0.7, 0.8)
AUC: 0.835
(0.667-0.944)
CXCL10
1030(0.6, 0.9)
AUC: 0.754
(0.567-0.896)
IL-8
28.2(0.6, 0.7)
AUC: 0.684
(0.48-0.854)
HGF
1170(0.9, 0.7)
AUC: 0.759
(0.564-0.932)
IL-9
453(0.8, 0.6)
AUC: 0.701
(0.479-0.895)
CTACK
1410(0.7, 0.7)
AUC: 0.736
(0.545-0.878)
SCF
189(0.8, 0.7)
AUC: 0.767
(0.6-0.931)
True positive rate
False positive rate

**Figure S5.** ROC curves evaluated on the cytokines that are resulted significant in the sera of patients subdivided between Exitus Group and Good Prognosis Group. The cutoff values are evidenced by red circles
